# Menstrual health and hygiene amongst adolescent girls and women of reproductive age: a study of practices and predictors, Odisha, India

**DOI:** 10.1186/s12905-024-02894-7

**Published:** 2024-02-26

**Authors:** Nishisipa Panda, Shyama Desaraju, Rudra Prasad Panigrahy, Upasona Ghosh, Shipra Saxena, Pratibha Singh, Bhuputra Panda

**Affiliations:** 1 PHFI-Indian Institute of Public Health, Bhubaneswar, India; 2 UNICEF India, New Delhi, India; 3 Present Address: KIIT School of Public Health, KIIT Deemed to be University, Bhubaneswar, India

**Keywords:** Practice, Knowledge, Sanitary pads, Menstrual hygiene, Women’s health, Odisha, India

## Abstract

**Background:**

Menstruation is a major physiological change in a woman’s life, but lack of knowledge, poor practices, socio-cultural barriers, poor access to products and their improper disposal have significant consequences on health, dignity and well-being of women and adolescent girls.

**Objectives:**

This study aimed to assess the knowledge and practices related to menstrual health and hygiene amongst females of 10–49 years of age; explore the experiences and challenges of women during menstruation; and identify the key predictors of healthy menstrual health and hygiene.

**Methods:**

Using a cross-sectional study design, we adopted a mixed methods approach for data collection. For quantitative household survey, a total of 921 respondents were selected from three districts of Odisha. Qualitative findings through focus group discussions and in-depth interviews supplemented the survey findings and helped to identify the barriers affecting good menstrual practices. Epi data version 2.5 and R 4.2.2 was used for data entry and data analysis, respectively. Descriptive statistics was used to calculate proportion, mean and standard deviation; Chi square test was used to measure the association between categorical variables. Bivariate and multivariate logistics analyses were done to identify predictors of healthy menstrual health and hygiene. For qualitative data analysis, thematic analysis approach was adopted using software Atlas.ti 8.

**Results:**

For 74.3% respondents, mothers were the primary source of information; about 61% respondents were using sanitary pad. The mean age at menarche was 12.9 years and almost 46% of respondents did not receive any information about menstruation before menarche. Lower age and education up to higher secondary level or above had statistically significant associations with the knowledge about menstruation. Age, caste, respondent’s education, mother’s education, sanitation facility, availability of water, accessibility and affordability for sanitary pads were found to be strongly associated with good menstrual hygiene practices.

**Conclusion:**

Traditional beliefs regarding menstruation still persists at the community level. Educating mothers, increasing awareness about safe menstrual hygiene, providing adequate water and sanitation facilities and ensuring proper disposal of menstruation products need priority attention.

## Introduction

Menstruation is a physiological process for women of reproductive age. On an average a woman cumulatively menstruates for about five to seven years during her lifetime, thus menstrual health and hygiene constitutes a critical life event. Every woman has the right to healthy, safe and dignified menstruation. However, evidences indicate this basic human right of women is not being addressed adequately, as adolescent girls and women experience a multitude of personal challenges and cultural barriers to a safe and comfortable menstruation. Poor knowledge, inadequate clean water and soap, insufficient infrastructure, non-availability of private places to clean and change the products, and poor awareness about disposal of used products are but few of those [1–4]. Moreover, poor social support systems, fear, stigma, uncertainty, mis-information at family and societal levels and personal inhibitions often act as barriers to managing safe menstruation. Further, due to cultural practices and restrictions many girls are not adequately informed about healthy menstruation around which exist many a myths, taboos and stigma [5].

Over the years, women have used some strategies to cope with menstruation, varying greatly across settings, based upon personal preferences, resource availability, cultural beliefs, social norms, socio economic status, and access to information. In many low-middle-income countries (LMIC), women and girls have restricted mobility and behaviour during menstruation as they are considered to be “impure” during menstruation. In developing countries menstruation is still often shrouded in secrecy, and mothers are reluctant to discuss menstruation with their daughters mainly due to lack of knowledge about menstruation [6].

In India, although menstruation is considered a natural event, “a gift from God”, women’s perception of it varies significantly across cultures and religions [7]. Limited knowledge and too many misconceptions about menstruation is mostly passed on from the mothers to the young girls as seen in parts of Rajasthan [8], Gujarat [9], Haryana [10], and Kerala [11]. This usually leads to undue fear, anxiety, and undesirable practices amongst young girls and women. Studies indicate the knowledge and practices related to menstruation are dependent on socio economic conditions as well [12]. Non-availability of and unaffordability to buy readymade sanitary napkins, especially in rural areas, is found to be associated with use of old cloth, homemade napkins, and cotton wool products [11].

MHH being an important public health concern, considerable research has been done so far in this field. In Odisha state, the focus of menstrual health and hygiene (MHH) research is centered around adolescent girls in school settings; and considerably less attention has been given to women’s experience of MHH at other life stages. Some studies have also aimed to focus on menstruation-related experiences at the workplace and in emergency situations [13, 14]. However, there is scant evidence on holistic understanding of the practices and the predictors of menstrual health. This study specifically aimed to assess the knowledge, practices and predictors of MHH in Odisha.

The specific objectives were:

1. To assess the knowledge and practices related to menstrual health and hygiene amongst females of 10–49 years of age.
2. To explore the experiences and challenges of women during menstruation.
3. To identify the predictors of healthy menstrual health and hygiene.

## Methods

### Study settings

Odisha has a population of about 45 million out of which about 33% live in poverty [15, 16]. With weak infrastructure compared to other states, low socio-economic status, and high levels of fertility the state has been included along with seven other EAG states. Approximately 40% of the state’s population does not have improved sanitation in their homes, and nearly 10% of households do not have piped water supply into their dwelling/plot [17]. The state has high gender disparities, including less access to economic opportunities for women compared to other low-income states; and in most households, males hold the power for sanitation-related decisions [15–17]. According to NFHS-5, about 81.5% of women in the age-group of 15–24 years use some method of ‘hygienic menstrual protection’ (e.g., locally prepared napkins, sanitary napkins, and tampons); however, comparable information is not available for women of other age groups [17]. Prioritizing menstrual health and hygiene for adolescent girls, in 2018, the state government launched a free sanitary napkin distribution program named KHUSHI for school going girls of 6th -12th standard. We conducted the study using a mixed-method approach and collected data from three sample districts of Odisha (Bhadrak, Koraput and Balangir) during September-November 2022. These districts were purposively selected in due consultation with the government officials so as to have urban, rural and tribal representation in the sample. Both quantitative (household survey) and qualitative (in-depth interview (IDI), focus group discussion (FGD) and key informant interview (KII) techniques were applied for data collection and analysis.

### Sampling and sample size

**Fig. 1:**
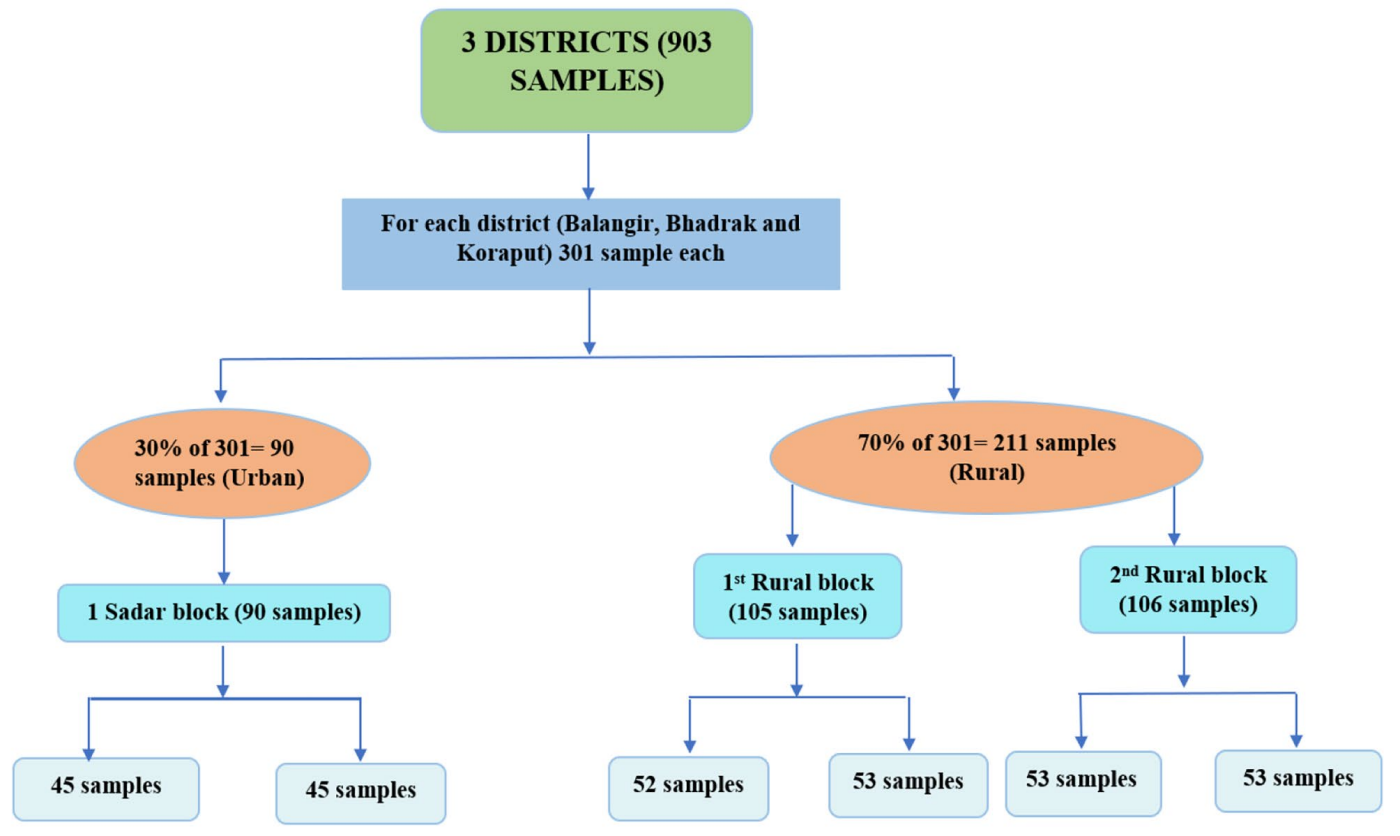
Sampling method and sample size allocation

The sampling for household survey was done assuming a 34% [18] prevalence of the rarest of the rare measures (such as prevalence of use of sanitary napkins among menstruating women of Odisha), 5% acceptable margin of error for 95% confidence interval, and a design effect of 2 (to account for clustered nature of the data): the calculated sample size was estimated at 690. With a non-response rate of 10%, the sample size increased to 759. Given that the mean age of menarche in India has been decreasing over the years [19, 20], and that the KHUSHI scheme is being implemented among adolescents in the 6th to 12th grades, we wanted to assess the MHH status and challenges among these girls in the early adolescent age group as well. Moreover, according to 2011 Census data, the 10–14 age group accounted for around 10% of female population of Odisha, while 15–49 age group accounted for 53% of total female population; the former group is about one-fifth the size as the latter. Thus, a total of 903 sample was estimated, against which we actually ended up collecting data from 921 respondents just to compensate for any missing data (Fig. 1).

### Quantitative data collection

The survey was conducted in the sample villages using a semi-structured interview schedule. Questions were asked to examine the knowledge, practices and challenges of girls/women in terms of accessibility to products and waste disposal methods. Basic demographic and socioeconomic background of individuals was collected which helped us in identifying the predictors. Key variables included: age of menarche, duration of bleeding, source of information, and experiences during menstruation. Demographic data included: age, caste, religion, occupation, educational qualification of the respondent and the parents, socioeconomic class, monthly income of the family, knowledge about menstruation, and practices with regard to of use of products, disposal of used products, myths and social stigma, challenged experienced during menstruation.

### Qualitative data collection

The main purpose of qualitative analysis was to (a) triangulate quantitative analysis, and (b) explore/explain beliefs, taboos, practices and factors contributing to menstrual health and hygiene. Twelve In-depth interviews (IDI) were conducted with eligible women participants from rural (village) and an urban (slum) settings. Six FGDs, comprising of 6–7 members, each, were conducted with adolescent girls (school-going and non-school-going), and adolescent boys (school-going): both tools aimed to explore the understanding, practices and challenges specific to menstrual health and hygiene. Thirty Key Informant Interviews (KII) were conducted with officials of the health and family welfare department, district administration, block administration, school teachers and front-line health workers as to explore their perspectives on menstrual health and hygiene, preparedness of the system to implement KHUSHI and the systemic bottlenecks. Key findings are presented in a separate paper.

Tailor-made interview guides (IDI/KII) and a topic guide (FGD) were meticulously developed, translated in to local language and field-tested before use. All the study tools were developed through iterative contextualization.

Informed consent was obtained from each of the participants and/or guardians before the interview started. Participation in the study was voluntary; confidentiality of information was maintained by omitting any personal identifier from the schedule. Respondents were informed of their full rights to skip or ignore any question or withdraw from their participation at any stage.

### Data quality assurance

The core team of researchers conducted daily debriefing sessions with the field investigators (FI) and interviewers. Two researchers were dedicatedly assigned to data cross-checking and cleaning on a daily basis. In case of any discrepancies, the concerned FI was consulted for immediate correction. During IDI and FGD, other than the interviewer, a field assistant was noting down some of the critical information across data collection sites. An iterative approach was followed by which each and every interview and focus group discussion was re-visited before moving to the next. The focus group discussion findings were summarized towards the end and validated by participants. The verbatim transcripts were read over and checked along with the audio track to nullify discrepancies.

### Data management and analysis

For the determination of the impact of socio-economic parameters on the Knowledge and practice a mean knowledge and practice score was obtained using Principal Component Analysis (PCA). The questions on knowledge dimension were: *cause of menstruation; organ where menstrual blood comes from; knowledge about menstruation before attaining menarche; whether or not having prior knowledge about menarche helpful; whether or not educating girls and boys about menarche important; good menstrual hygiene practices; whether or not poor menstrual hygiene practices lead to infection; duration of normal menstrual cycle; interval between menstrual cycles; age for menopause; and whether or not menstruation affects child-bearing.* Questions on practice included: *type of menstrual absorbents used; outing habits during menstruation; frequency and place of changing the menstrual absorbent; hand-washing before and after changing; frequency of washing genitals during menstruation; disposal site of the used menstrual absorbent; and whether or not the menstrual absorbents were wrapped up before disposing*. Each response on knowledge and practice was assigned a particular weightage, obtained through PCA. Score of 1 and 0 assigned to correct and incorrect/don’t know responses, respectively. Practice-related responses were scored in an ordinal scale: the most appropriate response received 5, and the least received zero. In some instances, scores ranged from 0 to 3. Since the measuring scales were different and weightage of questions varied, thus, through PCA we scored a set of thirteen knowledge questions and twelve practice questions and then used the first eigenvalue to construct tertiles of knowledge and practice index. Thus, we created three different groups of ‘poor’, ‘average’ and ‘good’ knowledge/practice having an equal representation of individuals in each category.

The household survey was conducted by six female interviewers who were trained by the principal investigator and the core team of researchers. Electronic devices were used for data collection through epi info software version 7.2.5; while software R version 4.2.2 was used for data analysis. Descriptive statistics was used to calculate proportion, mean and standard deviation. Chi square test was used to measure the association between categorical variables. Bivariate and multivariate logistics analyses were done to identify factors associated with knowledge and practices of menstrual health.

The qualitative data were translated and transcribed, verbatim, by the research team and then imported to Atlas.ti 8 for analysis. Thematic analysis approach was adopted for data analysis [21]. The steps of analysis included: data familiarisation, coding and thematic grouping for interpretation of results. The results section contains both quantitative and qualitative findings.

## Results

The results are presented under specific themes: [1] socio-demographic profile, [2] knowledge and preparedness related to menstruation; [3] practices related to menstruation; [4] experiences and challenges related to menstruation; and [5] predictors related to menstruation.

### Socio-demographic profile

All respondents (*n* = 921) who participated in the survey were females in the age group of 11–49 years, with mean age of 25 years. Most of the participants were Hindus (96%) followed by Christians (2.2%) and Muslims (1.6%). In terms of education, majority of the participants (50.4%) had completed secondary standard (Grade 6–10), followed by higher secondary and above (27.8%); 6.8% were in primary standard (Grade 1–5), while 15% participants didn’t have any formal education. Majority of the study participants were from Other Backward Classes [(OBC), (42.3%)], followed by Scheduled Tribes (21.8%), Scheduled Castes (17.7%) and General (17%) caste. About 24.6% of the participants were pursuing education, 17.3% were employed and 58.1% respondents were unemployed. Among those employed (*n* = 159), 30.2% were self-employed in farming, 29.6% were daily wage labourers, 23.9% were self-employed in non-farming sector, and 16.4% were salaried. About 74% of the respondents came from nuclear families, while 26% were from joint families. With regard to toilet/sanitation facilities at home 54.3% of participants used pit latrine, 41.4% participants used open ground, 2.4% used flush latrine and 1.8% visited community pit latrines. In terms of source of non-drinking water, majority (31.4%) of participants obtained it from bore well or hand pump, 28.3% from in-house taps, 17.3% brought from river/pond, 13.5% sourced water from community taps, while the remaining 9.6% participants got it from dug well (Table 1).

Fathers/husbands of 39.7% respondents had completed secondary-level education; 24.6% had completed higher secondary education; 13.8% had completed primary school; and 21.8% were illiterate. On the contrary 51.7% mothers were illiterate, only 4.3% had completed higher secondary education, 23.2% had completed secondary-level education while 20.7% had completed primary education (Table 1).

### Knowledge about menstruation

#### Quantitative findings

About 74.3% respondents knew that menstruation is a physiological process, while 14.4% were unaware of its aetiology. Around 6.7% participants said menstruation is a “Curse of God” while 3.9% people mentioned it is caused by a disease. The primary source of information about menstruation for most participants was their mothers (74.3%), followed by other family members (8.9%), friends (7.8%), sisters (4.7%) and teachers (4.2%). Around 18.2% respondents mentioned that menstruation is still considered a ‘secret’ in their locality. The reasons cited were: shyness (73.2%), too personal a concept (17.9%), stigma (7.1%) and religious sanctions (1.7%). About 46.9% respondents did not know about menstruation before menarche. About 67.2% of the girls and women got scared when they experienced their first periods, 19.5% were embarrassed, 3.4% were guilty while 9.9% had no reaction at all. About 62.6% stated to experience normal periods while the rest 37.4% experienced some symptoms/difficulties – amongst these, almost 60% didn’t seek any external care, 18.6% consulted with an ASHA (Accredited Social Health Activist) worker or a doctor, 12.5% respondents discussed with their mothers, while 9% respondents took some home remedies (Table 2).

#### Qualitative findings

Qualitative findings revealed that many girls and women did not have prior knowledge about menstruation before attaining menarche. Because of lack of awareness and preparedness, almost everyone found the very first experience of seeing a bloodstain *“scary”.* None of the participants had knowledge about the physiological reasons of menstruation, and they explained the process as:

*“Loss of bad blood from the body and formation of new blood”*. Most of the girls didn’t have an idea about the source of menstrual blood and said they were *“too scared to check”* (FGD Girls, Bhadrak).

Most women felt the need and importance of knowing about menstruation before menarche, but this was not translated into action as they themselves had not prepared their daughters for this experience. They reasons cited were “today’s girls know everything”, “felt shy to discuss”, and “it’s too early”. The school-going girls opined that it is important to educate girls on menstruation before they reach that stage in life for better preparedness.

*“If the girls knew about this early, they will be aware and not be scared, and they can carry clothes or pads along with them wherever they go” [IDI mother, Koraput]*.

**Table 1:** Socio-demographic details of the respondents (*n* = 921)

Categories	Sub-categories	Total (*n* = 921)
**Age**		25.32 (± 8.86)
	10–19 years	290 (31.5%)
	20–29 years	332 (36%)
	30–39 years	219 (23.8%)
	40–49 years	80 (8.7%)
**Religion**		
	Hindu	886 (96.2%)
	Christian	20 (2.2%)
	Muslim	15 (1.6%)
**Education**		
	Illiterate	138 (15.0%)
	Primary	63 (6.8%)
	Secondary	464 (50.4%)
	Higher Secondary and above	256 (27.8%)
**Caste**		
	General	157 (17.0%)
	Other Backward classes	390 (42.3%)
	Scheduled Caste	163 (17.7%)
	Scheduled Tribes	201 (21.8%)
	Others	10 (1.1%)
**Employment**		
	Employed	159 (17.3%)
	Pursuing education	227 (24.6%)
	Unemployed	535 (58.1%)
**Occupation (n = 159)**		
	Daily wage laborer	47 (29.6%)
	Regular salaried	26 (16.4%)
	Self-employed in farm	48 (30.2%)
	Self-employed in non-farm	38 (23.9%)
**Marital status**		
	Married	541 (58.7%)
	Unmarried	366 (39.7%)
	Divorced/Separated/Widow	14 (1.5%)
**Educational qualification of father/Husband**		
	Illiterate	201 (21.8%)
	Primary	127 (13.8%)
	Secondary	366 (39.7%)
	Higher Secondary and above	227 (24.6%)
**Educational qualification of mother**		
	Illiterate	476 (51.7%)
	Primary	191 (20.7%)
	Secondary	214 (23.2%)
	Higher Secondary and above	40 (4.3%)
	Others	15 (1.6%)
**Dwelling**		
	Kaccha	190 (20.6%)
	Semi-Pucca	351 (38.1%)
	Pucca	380 (41.3%)
**Wealth Index**		
	Poor	320 (34.7%)
	Middle-class	298 (32.4%)
	Rich	303 (32.9%)
**Sanitation facility**		
	Open area (ground)	381 (41.4%)
	Community pit latrine	17 (1.8%)
	Own toilet	522 (56.7%)
	Others	1 (0.1%)
**Source of non-drinking water**		
	Community Tap	124 (13.5%)
	Dug well	88 (9.6%)
	Hand pump/ Bore well	289 (31.4%)
	Spring/river/pond	159 (17.3%)
	Tap inside residence	261 (28.3%)

Often the information about menstruation that was transmitted down through family members (including through mothers and elder sisters) was about the various socio-cultural customs associated with menstruation and the possible negative consequences of not adhering to it. The girls, after attaining menarche, were told by their mothers they attained *“adulthood”* and therefore had to live by certain rules. In Koraput district, most girls were aware about menstruation prior to menarche and were prepared for it. They explained about the unique tradition of organizing community feasts to celebrate attainment of menarche; this served as a source of information about menstruation for younger girls who were better prepared as compared to girls of other districts wherein such explicit information transmission was not in vogue.

*“When my elder sister attained menarche, a community feast was organized to celebrate the event, from there I knew such thing will happen to me as well” (FGD Girls, Koraput).*

Discussions with adolescent boys revealed that a majority of them learned about menstruation by watching their mothers or sisters adhere to certain rules. They had no idea about what women and girls go through during menstruation or the physiological process that causes it, nor did they have much understanding of the physical and emotional discomfort associated with it. They were aware that other girls in their class were going through the same thing, but viewed it as something mysterious, to be discussed only in secrecy.

*“At home they sit in a separate/secluded place and they do not touch anything during that time, whatever they require we give. The ladies at our home have this, so we know. They get stomach pain…. Don’t like to eat food at that time…. they feel weak” [FGD Boys, Koraput].*

### Practices related to menstruation

#### Quantitative findings

About 61% respondents used only sanitary pads at home as a menstrual absorbent material, while 31.6% used only cloth, and 7.2% used both sanitary pads and cloth. This pattern was similar even during travelling out (66%: sanitary pads; 28%: cloth; 4.5%: sanitary pads and cloth). Use of only cloth as a menstrual absorbent was higher in the older age group as compared to the younger generation. Further, about 21.4% participants mentioned that they were not comfortable traveling during periods (Table 2).

**Table 2:** Information and restrictions during menstruation

Category	Sub category	Total (*n* = 921)
**Cause of Menstruation**		
	Normal physiological process	684 (74.3%)
	Caused by disease	36 (3.9%)
	Curse of God	62 (6.7%)
	Don’t know	133 (14.4%)
	Others	6 (0.7%)
**Source of Information**		
	Mother	684 (74.3%)
	Sister	43 (4.7%)
	Other family member/relative	82 (8.9%)
	Friends	72 (7.8%)
	School teachers	39 (4.2%)
	FHW	1 (0.1%)
**Menstruation is a secret**		
	No	753 (81.8%)
	Yes	168 (18.2%)
**Reason (** *n* ** = 168)**		
	It’s personal/Normal	30 (17.9%)
	Religion	3 (1.8%)
	Shyness	123 (73.2%)
	Stigma	12 (7.1%)
**Knowledge about Menarche**		
	No	432 (46.9%)
	Yes	489 (53.1%)
**Reaction to first menstruation**		
	Scared	619 (67.2%)
	Embarrassed	180 (19.5%)
	Guilty	31 (3.4%)
	No reaction	91 (9.9%)
**Reaction to not getting periods on time**		
	Relieved/ Happy	64 (6.9%)
	Scared	65 (7.1%)
	Worried	345 (37.5%)
	Has never happened	204 (22.1%)
	No reaction	243 (26.4%)
**Experience of Periods**		
	Normal	577 (62.6%)
	Experience mild symptoms	270 (29.3%)
	Experience severe symptoms	58 (6.3%)
	Severe bleeding/Unusual discharge	16 (1.7%)
**Seeking care during Periods (** *n* ** = 344)**		
	Didn’t do anything	205 (59.6%)
	Did home remedies	31 (9.0%)
	Consulted ASHA/Doctor	64 (18.6%)
	Discussed with mother	43 (12.5%)
	Others	1 (0.3%)
**Restrictions during Periods**		
	Not go to certain places	133 (14.4%)
	Not allowed to touch certain things	271 (29.4%)
	Avoid certain food	69 (7.5%)
	Not allowed to cook	299 (32.5%)
	Not allowed to go out	113 (12.3%)
	Not allowed to attend religious activities	588 (63.8%)
	Not allowed to sleep with other family members	258 (28.0%)
**Access to sanitary pads in your locality**		
	No	167 (18.1%)
	Yes	754 (81.9%)
**Able to afford sanitary pads**		
	No	341 (37%)
	Yes	580 (63%)

#### Qualitative findings

Most of the women and girls said they used cloths during menarche due to non-availability of pads and compliance with the advice of their mothers. However, girls later switched to pads when they learnt about it from school based- KHUSHI program. Most of the middle-aged women, even though were aware about the usage of sanitary napkins, they preferred to use cloths to absorb menstrual blood as they had already been adapted to it and some of them also mentioned about financial hardships in buying pads. Some believed cloths have had better absorbent capacity compared to commercial sanitary napkins.

*“I don’t feel comfortable with pads. I get heavy bleeding, so I don’t feel comfortable and safe using pads” [IDI mother, Balangir]*.

Choice of menstrual absorbent was mostly driven by an individual’s perception about the soaking capacity and efforts required to maintain it, as shared by a woman from Bhadrak,

*“For cloths, we need to clean with Dettol and dry it at a clean place, but after using pads, I don’t have to face such hassles. I just need to remove it and then wash hands properly” [IDI Mother, Bhadrak]*.

Most of the girls and women reported bathing in the backyard of the house where a bucket of water is given to them by other family members.

*R1: “We take water to another place and bath there”.*

*R2: “We are not allowed to bath at the same place”.*

*R3: “I take bath at the backyard of our house”.*

*(FGD girls, Balangir)]*

Majority of the women reported changing the cloth twice a day, i.e., in the morning and evening. The girls although were aware that *a pad should be changed every 2–3 h*, but in practice they changed it twice or thrice a day.

*“It depends on the bleeding; if bleeding is less, then you need to change twice in a day. But if bleeding is more, then you need to change it every two or three hours” [IDI mother, Bhadrak]*.

**Table 3:** Bivariate analysis of factors associated with knowledge of menstruation

	Poor (*n* = 307)	Average (*n* = 307)	Good (*n* = 307)	Total (*n* = 921)	*p* value
**Age**					0.254
10–19 years	98 (33.8%)	98 (33.8%)	94 (32.4%)	290 (100.0%)	
20–29 years	96 (28.9%)	115 (34.6%)	121 (36.4%)	332 (100.0%)	
30–39 years	78 (35.6%)	70 (32.0%)	71 (32.4%)	219 (100.0%)	
40–49 years	35 (43.8%)	24 (30.0%)	21 (26.2%)	80 (100.0%)	
**Caste**					**< 0.001**
Scheduled Tribes	65 (32.3%)	92 (45.8%)	44 (21.9%)	201 (100.0%)	
Scheduled Caste	67 (41.1%)	52 (31.9%)	44 (27.0%)	163 (100.0%)	
Other Backward classes	124 (31.8%)	116 (29.7%)	150 (38.5%)	390 (100.0%)	
General	45 (28.7%)	46 (29.3%)	66 (42.0%)	157 (100.0%)	
Others	6 (60.0%)	1 (10.0%)	3 (30.0%)	10 (100.0%)	
**Region**					0.026
Urban	78 (28.5%)	88 (32.1%)	108 (39.4%)	274 (100.0%)	
Rural	229 (35.4%)	219 (33.8%)	199 (30.8%)	647 (100.0%)	
**Education of the respondent**					**< 0.001**
Illiterate	62 (44.9%)	55 (39.9%)	21 (15.2%)	138 (100.0%)	
Primary	19 (30.2%)	25 (39.7%)	19 (30.2%)	63 (100.0%)	
Secondary	146 (31.5%)	156 (33.6%)	162 (34.9%)	464 (100.0%)	
Higher Secondary and above	80 (31.2%)	71 (27.7%)	105 (41.0%)	256 (100.0%)	
**Education of the mother**					0.036
Illiterate	158 (33.2%)	179 (37.6%)	139 (29.2%)	476 (100.0%)	
Primary	62 (32.5%)	55 (28.8%)	74 (38.7%)	191 (100.0%)	
Secondary	72 (33.6%)	66 (30.8%)	76 (35.5%)	214 (100.0%)	
Higher Secondary and above	15 (37.5%)	7 (17.5%)	18 (45.0%)	40 (100.0%)	
**Wealth Index**					0.028
Poor	119 (37.2%)	112 (35.0%)	89 (27.8%)	320 (100.0%)	
Middle-class	92 (30.9%)	107 (35.9%)	99 (33.2%)	298 (100.0%)	
Rich	96 (31.7%)	88 (29.0%)	119 (39.3%)	303 (100.0%)	

Respondents said they maintained hygiene during periods with the help of soap and Dettol. The cloth-users used old *Sarees*, towels, etc. as absorbents and washed those cloths using hot water and Dettol. They also dried the cloth under direct sunlight and reused the same cloth for almost 5–6 months.

*“I wash the cloth with Dettol water and dry it under direct sunlight. After using it for six months, I burn those cloths and use another set of cloths. At the end of cycle, I dip the cloth in hot water, clean it nicely and dry it under direct sunlight. Once it dries, I keep it in a polythene bag and store it for next month” [IDI mother, Balangir]*.

The disposal practices constituted washing the pads and cloths prior to disposing of them. Some people kept their pads in a polythene and stored them at a place until their period ends, and then disposed those off at one go. The usual means of disposal in rural areas included throwing used pads and cloths in open or in the pond, burying it in the backyard, burning it by petrol/kerosene, and flushing it out in the toilets. Majority of the girls in the FGDs mentioned that they flushed the pads in the toilets at home or in school, being unaware of the consequence of doing so. Urban respondents used polythene bags to dispose of pads in the municipality waste collection van. Some of them also threw sanitary materials in the community dustbins.

*“Since this is a village area, people don’t throw used absorbents as such, they wash and then dispose it. Either they dig a hole near the pond and bury it, or they burn it. I keep them in the polythene bags until my cycle ends and then bury it. Village women are very careful about this as it is believed that if a snake crawls over the pad, or is eaten by mistake by cows or goats while grazing, then girl/women will be cursed. So, either it is buried or burnt.” [IDI mother, Balangir]*.

**Table 4:** Multivariable analysis of factors associated with knowledge of menstruation

	OR	95%CI	AOR		95%CI
**Age**					
10–19 years	1.45	(0.91–2.31)		1.44	(0.86–2.39)
20–29 years	1.77	(1.12–2.80)		1.79	(1.11–2.89)
30–39 years	1.39	(0.86–2.25)		1.52	(0.93–2.48)
**Caste**					
Scheduled Caste	0.89	(0.61–1.30)	0.67		(0.44–1.01)
Other Backward classes	1.45	(1.06–1.97)	1.15		(0.79–1.64)
General	1.69	(1.15–2.48)	1.27		(0.80–2.01)
**Respondent’s education**					
Primary	1.9	(1.11–3.26)	1.87		(1.06–3.26)
Secondary	2.05	(1.45–2.92)	1.89		(1.24–2.88)
Higher Secondary and above	2.39	(1.63–3.52)	2.11		(1.32–3.38)
**Education of Respondent’s mother**					
Primary	1.26	(0.92–1.72)	0.89		(0.62–1.25)
Secondary	1.13	(0.84–1.53)	0.73		(0.50–1.04)
Higher Secondary and above	1.32	(0.70–2.49)	0.87		(0.43–1.73)
**Wealth Index**					
Middle class	1.29	(0.97–1.73)	1.14		(0.83–1.56)
Rich	1.47	(1.09–1.96)	1.16		(0.79–1.69)
**Region**					
Urban	1.42	(1.09–1.85)	1.29		(0.95–1.74)

*R1: I wash it and then throw it in the toilet and pour bucket full of water.*

*R2: We keep it at one place and at the end of the periods burry it in the backyard*.

*R3: Sometimes we flush it in the latrine and sometimes throw it in the pond” [FGD girls, Bhadrak]*.

**Table 5:** Bivariate analysis of factors associated with menstrual hygiene practices

	Poor (*N* = 307)	Average (*N* = 308)	Good (*N* = 306)	Total (*N* = 921)	*p* value
**Age**					< 0.001
10–19 years	51 (17.6%)	125 (43.1%)	114 (39.3%)	290 (100.0%)	
20–29 years	119 (35.8%)	102 (30.7%)	111 (33.4%)	332 (100.0%)	
30–39 years	97 (44.3%)	59 (26.9%)	63 (28.8%)	219 (100.0%)	
40–49 years	40 (50.0%)	22 (27.5%)	18 (22.5%)	80 (100.0%)	
**Caste**					< 0.001
Scheduled Tribes	127 (63.2%)	51 (25.4%)	23 (11.4%)	201 (100.0%)	
Scheduled Caste	59 (36.2%)	58 (35.6%)	46 (28.2%)	163 (100.0%)	
Other Backward classes	109 (27.9%)	135 (34.6%)	146 (37.4%)	390 (100.0%)	
General	11 (7.0%)	64 (40.8%)	82 (52.2%)	157 (100.0%)	
Others	1 (10.0%)	0 (0.0%)	9 (90.0%)	10 (100.0%)	
**Region**					< 0.001
Urban	28 (10.2%)	105 (38.3%)	141 (51.5%)	274 (100.0%)	
Rural	279 (43.1%)	203 (31.4%)	165 (25.5%)	647 (100.0%)	
**Education of the respondent**					< 0.001
Illiterate	121 (87.7%)	11 (8.0%)	6 (4.3%)	138 (100.0%)	
Primary	41 (65.1%)	11 (17.5%)	11 (17.5%)	63 (100.0%)	
Secondary	121 (26.1%)	181 (39.0%)	162 (34.9%)	464 (100.0%)	
Higher Secondary and above	24 (9.4%)	105 (41.0%)	127 (49.6%)	256 (100.0%)	
**Education of the mother**					< 0.001
Illiterate	255 (53.6%)	142 (29.8%)	79 (16.6%)	476 (100.0%)	
Primary	29 (15.2%)	84 (44.0%)	78 (40.8%)	191 (100.0%)	
Secondary	18 (8.4%)	72 (33.6%)	124 (57.9%)	214 (100.0%)	
Higher Secondary and above	5 (12.5%)	10 (25.0%)	25 (62.5%)	40 (100.0%)	
**Wealth Index**					< 0.001
Poor	176 (55.0%)	98 (30.6%)	46 (14.4%)	320 (100.0%)	
Middle-class	101 (33.9%)	101 (33.9%)	96 (32.2%)	298 (100.0%)	
Rich	30 (9.9%)	109 (36.0%)	164 (54.1%)	303 (100.0%)	
**Sanitation facility**					< 0.001
Open field	204 (53.5%)	134 (35.2%)	43 (11.3%)	381 (100.0%)	
Community toilet	2 (11.8%)	9 (52.9%)	6 (35.3%)	17 (100.0%)	
Own toilet	101 (19.3%)	164 (31.4%)	257 (49.2%)	522 (100.0%)	
Others	0 (0.0%)	1 (100.0%)	0 (0.0%)	1 (100.0%)	
**Source of water (for household purposes)**					< 0.001
River/pond	113 (71.1%)	43 (27.0%)	3 (1.9%)	159 (100.0%)	
Hand pump/well	135 (35.8%)	138 (36.6%)	104 (27.6%)	377 (100.0%)	
Community Tap	26 (21.0%)	56 (45.2%)	42 (33.9%)	124 (100.0%)	
Tap inside residence	33 (12.6%)	71 (27.2%)	157 (60.2%)	261 (100.0%)	
**Access to sanitary pads in your locality**					< 0.001
No	100 (59.9%)	40 (24.0%)	27 (16.2%)	167 (100.0%)	
Yes	207 (27.5%)	268 (35.5%)	279 (37.0%)	754 (100.0%)	
**Able to afford sanitary pads**					< 0.001
No	190 (55.7%)	92 (27.0%)	59 (17.3%)	341 (100.0%)	
Yes	117 (20.2%)	216 (37.2%)	247 (42.6%)	580 (100.0%)	

### Experiences and challenges during menstruation

#### Quantitative findings

Almost 68% participants stated to have faced restrictions during menstruation; 14.4% of respondents claimed that they were restricted from visiting certain places, while 63.8% of respondents said they were prevented from participating in any religious activities. About 7.5% participants reported that they were told to avoid certain types of food; 12.3% of participants were prohibited from leaving the house, while 32.5% were debarred from cooking. About 18% of respondents stated that they had difficulty accessing sanitary pads in their locality, and 37% claimed they couldn’t afford to purchase sanitary pads. (Table 2)

#### Qualitative findings

Women and girls shared how their mothers provided the, with an exhaustive list of “don’ts” when they experienced menarche. They described this process of transition into adulthood as a distressing experience due to the prevailing cultural practices such as isolation, movement restrictions, and community feasts organized after 7 days of menarche. Although some restrictions were eased during subsequent periods, complete seclusion for one week was still observed during menarche in all three study sites. Menarche is considered a period of pollution, which is why girls cleanse themselves, clean the isolation area, have a full-body bath, wear new clothes and visit temple and seek blessings from elders on the seventh day. Some communities organized feasts after this.

*“I had to be confined to home for 7 days and was not allowed to touch anything/anyone, nor was anyone allowed to touch me. I was restricted from coming face to face with male members. I was warned if I disobey these rules, then something unfortunate might happen, and I would be responsible for bringing misfortune. This is the tradition and if something goes wrong, then puja has to be done. The Pandit has to be informed about the time and the details of onset of menarche to prepare the astrological chart, which is then followed to fix marriage” [IDI mother, Balangir]*.

### Predictors of knowledge and practices related to menstruation

Bivariate and multivariate logistic analyses revealed that respondents aged 20–29 years of age [AOR-1.79, 95% CI (1.11–2.89)] and educated to higher secondary level [AOR = 2.11, 95% CI (1.32–3.38)] had statistically significant associations with menstruation knowledge (Tables 3 and 4).

**Table 6:** Multivariable analysis of factors associated with menstrual hygiene practices

	OR	95%CI	AOR	95%CI
Age				
10–19 years	3.06	(1.92–4.93)	5.31	(2.87–9.89)
20–29 years	1.82	(1.15–2.92)	2.83	(1.59–5.10)
30–39 years	1.32	(0.81–2.17)	2.19	(1.20–4.01)
**Caste**				
Scheduled Caste	3.10	(2.08–4.66)	1.44	(0.87–2.40)
Other Backward classes	4.70	(3.35–6.66)	2.41	(1.51–3.86)
General	10.49	(6.93–16.02)	3.04	(1.73–5.40)
**Education of the respondent**				
Primary	4.29	(2.08–9.00)	1.78	(0.78–4.11)
Secondary	20.62	(12.29–36.67)	4.72	(2.58–9.03)
Higher Secondary and above	43.24	(24.98–78.94)	5.82	(3.03–11.61)
**Wealth Index**				
Middle class	2.62	(1.94–3.56)	1.01	(0.69–1.46)
Rich	7.84	(5.72–10.81)	1.07	(0.69–1.69)
**Education of respondent’s mother**				
Primary	4.68	(3.39–6.49)	2.17	(1.47–3.21)
Secondary	8.82	(6.34–12.36)	2.43	(1.61–3.68)
Higher Secondary and above	9.72	(5.08–19.36)	1.33	(0.62–2.93)
**Region**				
Urban	3.87	(2.95–5.09)	0.94	(0.63–1.38)
**Sanitation facility**				
Community toilet	4.57	(1.93–11.03)	0.76	(0.27–2.14)
Own toilet	5.8	(4.45–7.60)	2.10	(1.47–3.00)
**Source of water (for household purposes)**				
Hand pump/well	5.03	(3.42–7.48)	1.78	(1.05–3.05)
Community Tap	8.26	(5.18–13.33)	3.59	(1.96–6.63)
Tap inside residence	21.05	(13.72–32.77)	5.86	(3.16–10.95)
**Access to sanitary pads in your locality**				
Yes	3.69	(2.65–5.18)	2.51	(1.62–3.92)
**Able to afford sanitary pads**				
Yes	4.37	(3.36–5.72)	2.64	(1.88–3.73)

Similarly, age, caste, education of the respondent, mother’s education, sanitation facility, availability of water, accessibility and affordability for sanitary pads were found to be strongly associated with menstrual hygiene practices (Table 5, and 6). Good menstrual hygiene practices involve changing pads/cloths every 4–6 h, washing reproductive organs with water (without soap), and disposing the menstrual products either by deep burial or through waste disposal system or incineration. Respondents in the age group of 10–19 years [AOR = 5.31, 95% CI (2.87–9.89)] were 5.31 times and those in 20–29 years age-group [AOR = 2.83, 95% CI (1.59–5.10)] were 2.83 times more likely to have good menstrual hygiene practices as compared to the older age group of 40–49 years. Respondents from the general caste [AOR = 3.04, 95% CI (1.73–5.40)] were 3.04 times more likely to have good menstrual hygiene practices as compared to Scheduled Tribes. The respondents who had completed higher secondary education or above [AOR = 5.82, 95% CI (3.03–11.61)] were 5.82 times more likely to have best practices as compared to the ones who had no education. Respondents whose mothers were educated up to secondary standards [AOR = 2.43, 95% CI (1.61–3.68)] were 2.43 times more likely to have better practices as compared to those who were who had no education. Respondents with better sanitation facility (own toilets) [AOR = 2.10, 95% CI (1.68–3.35)], were 2.1 times more likely to have better menstrual hygiene practices compared to those visiting open grounds. Respondents who had proper water supply and taps connected to their households [AOR = 5.86, 95% CI (3.16–10.95)] were 5.86 times more likely to have better menstrual hygiene practices as compared to the ones who didn’t have. Most importantly accessibility [AOR = 2.51, 95% CI (1.62–3.92)] and affordability for sanitary pads [AOR = 2.64, 95% CI (1.88–3.73)] were also significantly associated with good menstrual hygienic practices.

## Discussion

Slowly but surely, the mean age of menstruation in India is on the decline, as we found it to be 12.94 (± 1.2) years which is similar to another study conducted in Mangalore, Karnataka [22]. Further, this was reported to be 13.98 years, 12.21 years, and 12.99 years, respectively, in studies carried out by Kamath et al. (Manipal), Bachloo et al. (Haryana), and Gupta et al. in Rajasthan [23–25]. With a huge rural-urban divide in terms of socio-demographic characteristics and access to information, this becomes crucial for the programme planners for resource allocation and strategy formulation in implementing schemes/interventions. In terms of awareness, our study revealed that 46.9% of respondents were not aware about menstruation prior to attainment of menarche which is similar to a systematic review that indicated only half of girls in India were informed about menstruation prior to menarche [5, 18]. Further, mothers were the primary source of information about menstruation - this is in sync with other studies from India, Nepal, South Africa and Pakistan [26–28]. However, in Egypt a study by El-Gilany et al. revealed that mass media was the main source of information about menstrual hygiene, followed by mothers [29]. Another study conducted among tribal (Gujjar) adolescent girls brought out that friends were the main source of information regarding menstruation [30]. Almost 25% of participants had no knowledge of the cause of menstruation, indicating that proper knowledge of menstruation is still lacking.

About one-fifth of respondents mentioned that menstruation is still kept a ‘secret’ and two-third had scary menarche experience. Similar proportion of women also experience restrictions during periods. Other studies conducted in different parts of India like Nagpur, West Bengal also have come up with comparable findings [31, 32]. These issues need to be addressed through community-based engagement of key stakeholders including school teachers and village representatives.

Multivariate analysis in our study revealed that younger generation and education up to higher secondary level and above were significantly associated with better knowledge regarding menstruation. In a study conducted among Lucknow college students in north India, Absar Ahmad et al. [33] in 2020, discovered that father’s education, occupation and monthly family income were associated with good knowledge about menstruation. Further, age, caste, respondent’s education, mother’s education, sanitation facility, availability of water, accessibility and affordability for sanitary pads were found to be strongly associated to menstrual hygiene practices. Yohannes Habtegiorgis et al. [34] in their study among high-school girls in Northeastern Ethiopia in 2020 also had found that maternal education, age, knowledge, and discussing menstrual hygiene with friends were significantly associated with good menstrual hygiene practises.

As the society is evolving, so are the individual preferences. For instance, more and more women, especially of younger age group, are now shifting towards use of disposable pads for ‘ease’ and ‘comfort’. However, the health and environmental dimensions of disposal of such pads is being discussed and debated globally. Recent research also has voiced about safety concerns related to the materials being used as the main absorbent material in disposable pads. In view of the long-term commitment of the government, disposal of pads must be addressed through development of proper procedures and enforcement modalities need to be worked out by the nodal agencies. The pollution control boards, urban planning department and local bodies both at urban and rural areas may have a pivotal role to play.

## Conclusion

The process of natural transition from adolescence to adulthood among girls is often found to be associated with traumatic experiences. Role of gender sensitive resources, education at family, community and society level, orientation of law makers and demystification of notions by religious leaders could be helpful in addressing reclusive practices. Age, education (of self and of mother), and the availability of water, sanitation facilities, accessibility and affordability for sanitary pads are key predictors of MHH knowledge and practices. In other words, the non-health determinants of health have strong women’s health consequences. Therefore, reasonable resources of the government, especially the local self-governments, may have to be earmarked for establishing water, sanitation and hygiene (WASH)-friendly infrastructure at schools, educational set-ups and public places. Improving access to safe and affordable sanitary materials to manage their menstruation has the potentiality to decrease risk of reproductive tract infections in women. Multi-pronged interventions are needed to overcome the structural barriers, reduce information gaps, improve access to menstruating products and ensure effective disposal of menstrual absorbents. In Odisha, the health and family welfare department, panchayati raj department, school and mass education department and local bodies may channelize their energy in shaping an evidence-based MHH policy for optimal women’s health outcomes, in turn, achievement of sustainable development goals as well as the objectives of the national health policy 2017.

### Study limitations

The study was conducted in three districts; therefore, generalization of the findings may be done with caution. The situation in urban cities may be different. The identified predictors of knowledge and practices may not be the exhaustive list.

## Data Availability

The datasets used and/or analysed during the current study are available with the corresponding author, which will be shared as and when needed, as a supplementary file.
